# Developing a Health Risk Evaluation Method for Triple H

**DOI:** 10.3390/ijerph16071168

**Published:** 2019-04-01

**Authors:** Chien-Chih Wang, Cheng-Ding Chang, Bernard C. Jiang

**Affiliations:** 1 Department of Industrial Engineering and Management, Ming Chi University of Technology, New Taipei 24301, Taiwan; 2 Department of Industrial Engineering and Management, Yuan Ze University, Chung-Li 32003, Taiwan; Justin_Chang@unimicron.com; 3 Department of Industrial Management, National Taiwan University of Science and Technology, Taipei 10607, Taiwan; bcjiang@mail.ntust.edu.tw

**Keywords:** multi-diseases, common risk factor, kernel density function, health risk curve

## Abstract

The development of a health evaluation system from human-related data is an important issue in preventive medicine. Previously, most studies have focused on disease assessment and prevention in patients. However, even if certain risk factors are all within normal ranges, individuals may not necessarily be completely healthy. This study focused on healthy individuals to develop a new index to assess health risks; this index can be used for the prevention of multiple diseases in healthy people. The kernel density technique was proposed to estimate the distribution of common risk factors and to develop a health risk index. A dataset of hypertension, hyperlipidemia, and hyperglycemia (Triple H) data from the National Health Insurance Research Database in Taiwan was used to demonstrate the proposed analytical process. The results of risk factor changes after six weeks of exercise were used to calculate the health risk index. The results showed that the subjects experienced a 7.29% reduction in their health risk index after the exercise intervention. This finding demonstrates the potential impact of an important reference index on quantifying the effect of maintenance in healthy people.

## 1. Introduction

For a long time, the diagnosis and treatment of diseases have been critical aspects of medical development. Many scholars have used data mining techniques on medical data to analyze the relationships between disorders and the real causes of those disorders [1,2,3,4]. Additionally, intelligent medical systems have been developed to help doctors diagnose illnesses [5,6,7]. However, abnormalities in physiological indicators may be a gauge of not only one disease but of multiple diseases. Therefore, in recent years, determining the common risk factors and developing a predictor model for multiple diseases have become more important. Chang et al. [8] proposed a two-stage analysis procedure that used data mining techniques and mathematical approaches to determine the common risk factors (such as systolic blood pressure (SBP), triglycerides (TGs), uric acid, glutamate pyruvate transaminase, and gender) and predictive models for hypertension and hyperlipidemia. Medical decision systems, based on analyzing risk factors and predicting the functions of diseases, can help patients understand the risks of developing diseases and efficiently provide diagnostic references for medical personnel. In the current era of medicine, preventive medicine has gradually become more accepted. The motivation of this study was to propose a new health risk index to assess the health status of people from the perspective of preventive medicine. The rapid development of medical devices has made it easy to obtain information about physiological indicators. In the past, when all physiological indicators were within the standard range, the probability of illness was small and the patient was likely healthy. However, such information does not help the concept of preventive medicine. Therefore, a novel index must be developed to assess health status when there are changes in risk factors in healthy people. 

Certain health care practices have also recently become more accepted; for example, people have started to use exercise, meditation, and diet to control risk factors. Whelton et al. [9] studied the effect of aerobic exercise on blood pressure and found that SBP and diastolic blood pressure (DBP) decreased by an average of 3.84 and 2.58 mmHg, respectively, over a period of aerobic training in 2419 subjects aged between 21 and 79 years old. Additionally, Fagard [10] designed a one-year experiment to observe the effects of exercise and diet on blood pressure; SBP and DBP decreased by an average of 3.4 and 2.4 mmHg, respectively, in 68 subjects after the implementation of an exercise regimen. Fagard [10] also found that exercise combined with dietary control resulted in a more significant effect than only exercise or only dietary control. Stewart et al. [11] designed an exercise plan for 115 subjects aged between 55 and 75 years old who were divided into two groups: a control group and a group that exercised three times a week. The results demonstrated that exercise could improve physical fitness and decrease the risk of many physiological factors related to cardiovascular diseases and diabetes. Based on the above discussion, exercise maintenance can reduce the values of several risk factors. 

However, there has been little research on a method to evaluate the health status of a healthy individual when their risk factors change even after exercise maintenance. For example, hypertension patients are defined by an SBP of >140 mmHg or a DBP of >90 mmHg. When the SBP ranges between 120 and 135 mmHg, which is within the normal range, the resulting health effects may differ in different individuals. Vasan et al. found that in 9845 subjects with normal blood pressure, only 5.3% became hypertensive after four years; however, 17.6% of the subjects who developed hypertension originally had an SBP between 120 and 129 mmHg and a DBP between 80 and 84 mmHg [12]. The American Diabetes Association defines diabetes as a fasting blood glucose level of >126 mg/dL. The normal fasting blood glucose level for non-diabetics should range between 70 and 100 mg/dL. Although a fasting blood glucose level of <100 mg/dL is normal, a fasting blood glucose level between 100 and 125 mg/dL is considered pre-diabetic. Nichols et al. found that 8.1% of patients with fasting glucose levels between 100 and 109 mg/dL and 24.3% of those with levels between 110 and 125 mg/dL developed diabetes after an average of 41.4 months [13]. Therefore, risk factor values that are closer to the threshold will increase the risk of developing the respective disease. From the perspective of preventive medicine, understanding the degenerating state of healthy individuals will help prevent future disease.

Maintainability is widely used in industrial applications. Maintainability can be used to assess the life of a machinery or production system. The physical structures of the human body contain similar production systems; thus, an approach transferring the concepts of maintainability to human health assessment is the focus of this study. In practical applications, information on risk factors (such as blood pressure, heart rate, Electroencephalography (EEG), and Electromyography (EMG) can be collected quickly with the widespread use of home medical equipment. Therefore, in this study, a kernel density technique was used to develop a health risk index based on risk factors from human-related data. In practice, changes in health risk indicators can be used to establish an early warning mechanism. When the health risk indicator is gradually declining, the patient will still be considered healthy; however, if they fail to control the risk factor, they will likely develop the disease. Thus, a health risk index can estimate the health status of people and help them better understand their health conditions. Additionally, manufacturers should apply new techniques [14,15,16] to develop rapid physiological devices or sensors to measure most risk factors. For example, Zhang et al. presents state-of-the-art research progress on cardiovascular health informatics and focuses on three major challenges: unobtrusive and wearable multi-parameter sensors, fast multimodal imaging technologies, and novel multi-scale information fusion heart models [17].

**Figure 1:**
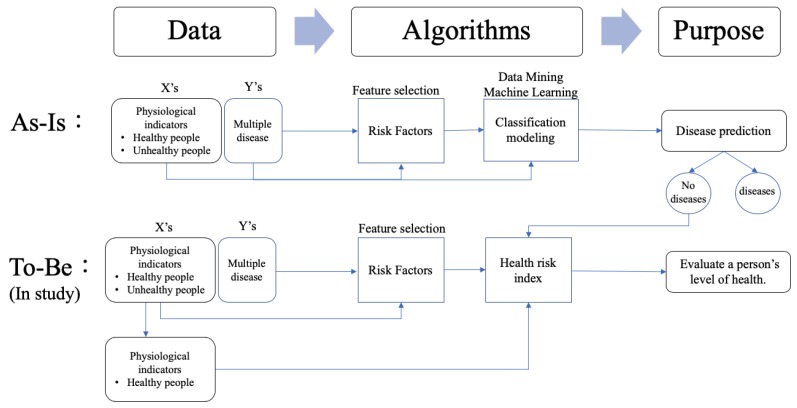
The research framework

In this paper, the health risk index was proposed to evaluate a person’s level of health. Figure 1 displays the framework of this study. The differences from previous methods are primarily a result of different study purposes. Previous research has developed disease prediction models through algorithmic designs by collecting information about specific diseases in healthy and unhealthy populations. Those algorithms use feature selection techniques to identify risks and establish a classification model by collecting data on risk factors. Their purpose is to determine whether a person is healthy or sick by measuring risk factors. Conversely, this study focuses on the assessment of healthy people. The similarity lies in the determination of risk factors, whereas the difference lies in the development of health risk indicators based on the data of healthy people. The purpose of this study was to assess health outcomes, particularly in high-risk groups. When health risk indicators decrease, individuals may develop diseases or otherwise become unhealthy. At this point, risk factors must be controlled to avoid disease. The results of this study can be used to jointly develop AI in healthcare through cloud computing to evaluate the trends and changes of users’ health. This information is important for disease prevention. 

## 2. Materials and Methods

This study proposed a novel three-stage analysis procedure involving the feature selection method, the kernel density estimation method, and mathematical approaches to calculate the health risk index of healthy people. Stage 1 adopted the findings from our previous study [8]. The procedure used six classification techniques to individually screen for the key risk factors for multiple diseases. Based on the results of previous research [8], we used five common risk factors: fasting plasma glucose (FPG), total cholesterol (T-CHO), TGs, SBP, and DBP, to determine the risks for hypertension, hyperlipidemia, and hyperglycemia. After identifying these risk factors, we compared the risk factors for each disease to determine the common risk factors for multiple diseases. Stage 2 used the kernel density estimation method to fit density curves for the common risk factors individually. Finally, stage 3 calculated the health risk index based on the kernel density function of the common risk factors. The two primary methods of kernel density estimation and the calculation of the health risk index are described below. The proposed methodology is explained below using data on 6496 subjects (3104 males and 3392 females) with Triple H disease from the National Health Insurance Research Database in Taiwan and the research results of Stewart et al. [11], who conducted a six-week exercise intervention. The reason for applying Stewart et al. [11] research data is that the five risk factors proposed are the same as the five common risk factors in this study. Also, the effectiveness of preventive maintenance was evaluated using a health risk curve.

### 2.1. Kernel Density Estimation Method

The kernel density estimation approach was first described by Rosenblatt [17] and Parzen [18]. It is a nonparametric statistical method used to estimate an unknown probability distribution. The method does not require a priori knowledge or make any additional assumptions regarding data distribution. In practice, it is often assumed that the values of the risk factor follow a normal distribution; however, this assumption lacks strong supporting evidence. Therefore, in this study, we adopted the kernel density method to estimate the risk factor distribution. 

If $x_{1},x_{2},\ldots ,x_{n}$ are independent and identically distributed unknown observations, the probability density function ${\hat{f}}_{h}$ can be estimated by the kernel density function as follows:

(1) $${\hat{f}}_{h}\left(x\right)=\frac{1}{nh}\sum_{i=1}^{n}K\left(\frac{x-x_{i}}{h}\right)$$

where K(⋅) is a kernel function that was symmetric and integrated into one. The h variable is the bandwidth to determine the degree of smoothness of the kernel function. This study evaluated six types of common kernel functions, including Gaussian, Epanechnikov, Triangular, Uniform, Bright, and Cosine, to estimate the kernel density values for all the common risk factors. Table 1 shows the six common types of kernel functions. 

When using the kernel density estimation method, we must choose the kernel function and set the bandwidth. The choice of kernel function was adopted through the six common types in Table 1. To select the kernel function, all values of the risk factors were initially used to calculate the probability density value as the real density value. Then, the six common types of kernel functions in Table 1 were used to estimate the kernel density function for the risk factor as the estimated density value. Finally, a suitable kernel function was chosen using the minimum difference between the sum of the real density value and the estimated density value. Therefore, each density function of the risk factors might be fitted with different kernel functions.

**Figure 2:**
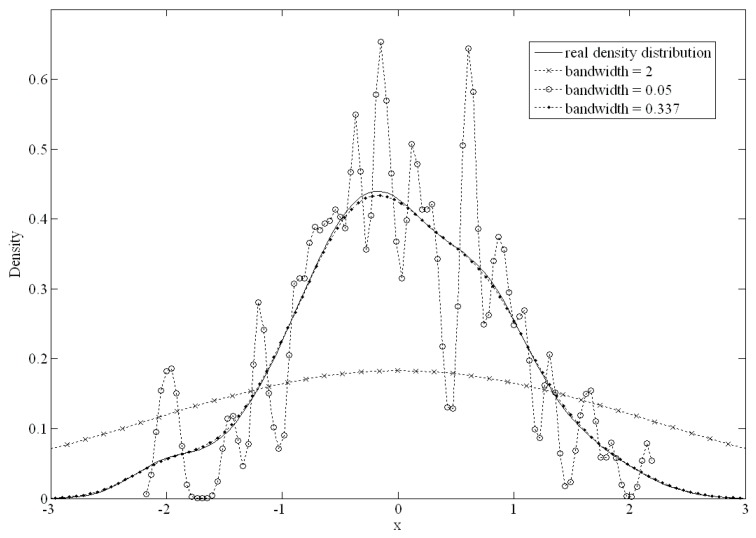
The differences with different bandwidth setting.

The bandwidth of the kernel function is an important parameter that has a strong influence on the resulting estimate. A narrow bandwidth would allow more over-fitting of the data. Conversely, an overly wide bandwidth would not have an appropriate data fit. Figure 2 shows the differences from 100 standard normally distributed datapoints. When the bandwidth was set to 0.05 or 2, there was a large difference from the original density distribution; conversely, if the bandwidth was set to 0.337, there was an excellent fit. 

The mean integrated squared error (*MISE*) was used to choose the best bandwidth. The *MISE* can be calculated as follows:

(2) $$MISE\left(h\right)=\int Bias^{2}\left({\hat{f}}_{h}\left(x\right)\right)dx+\int Var\left({\hat{f}}_{h}\left(x\right)\right)dx$$

The *MISE* was separated into two parts including $Bais\left({\hat{f}}_{h}\left(x\right)\right)=E\left[{\hat{f}}_{h}\left(x\right)\right]-f\left(x\right)$ and $Var\left({\hat{f}}_{h}\left(x\right)\right)=E\left[{\hat{f}}_{h}^{2}\left(x\right)\right]-E^{2}\left[{\hat{f}}_{h}\left(x\right)\right]$. ${\hat{f}}_{h}\left(x\right)$ was the estimated density value using the kernel function, and f(x) was the unknown true density value. $\int Bias^{2}\left({\hat{f}}_{h}\left(x\right)\right)dx$ using the Taylor expansion method was derived as $1/4\left[h^{4}\mu_{2}^{2}R\left(f\right)\right]$, $\mu_{2}=\int z^{2}K\left(z\right)dz$ and $R\left(f\right)=\int f^{\prime \prime }{\left(x\right)}^{2}dx$. *n* and *h* were the sizes of the data and bandwidth, respectively. If *n* was large and *h* was small, the variance of ${\hat{f}}_{h}\left(x\right)$ can be derived as $\left(1/nh\right)\int K^{2}\left(z\right)dz$. Therefore, an approximate mean integrated squared error (*AMISE*) was calculated as follows:

(3) $$AMISE=\frac{1}{4}h^{4}\mu_{2}^{2}R\left(f\right)+\frac{1}{nh}\int K^{2}\left(z\right)dz$$

We estimated the optimal bandwidth by minimizing *AMISE* regarding *h* by the first derivative. The optimal bandwidth *h* was:

(4) $${\hat{h}}_{AMISE}={\left[\frac{\int K^{2}\left(z\right)dz}{\mu_{2}^{2}R\left(f\right)}\right]}^{\frac{1}{5}}n^{-\frac{1}{5}}$$

In practice, the above formula has an infinite loop problem when calculating both $\int K^{2}\left(z\right)dz$ and an unknown function $f^{\prime \prime }\left(x\right)$. Liu et al. compared the accuracies of nine types of bandwidth selection methods and found that no particular method performed better for all problems [19]. Therefore, in this study, we used the NDR0 method, which was suggested by Liu et al. [19]. The NDR0 method has the advantage of being easily calculated using the standard deviation ($\hat{\sigma }$) and inter-quartile range of the dataset. The bandwidth can be calculated using the NDR0 as follows:

(5) $$\hat{h}=0.9\min\left(\hat{\sigma },\frac{IQR}{1.34}\right)n^{-\frac{1}{5}}$$

From the above procedure, we can determine the probability density function of the risk factors using the kernel density estimation approach. Next, the health risk index was proposed to estimate the health status of healthy people. 

### 2.2. Health Risk Index Calculation

**Figure 3:**
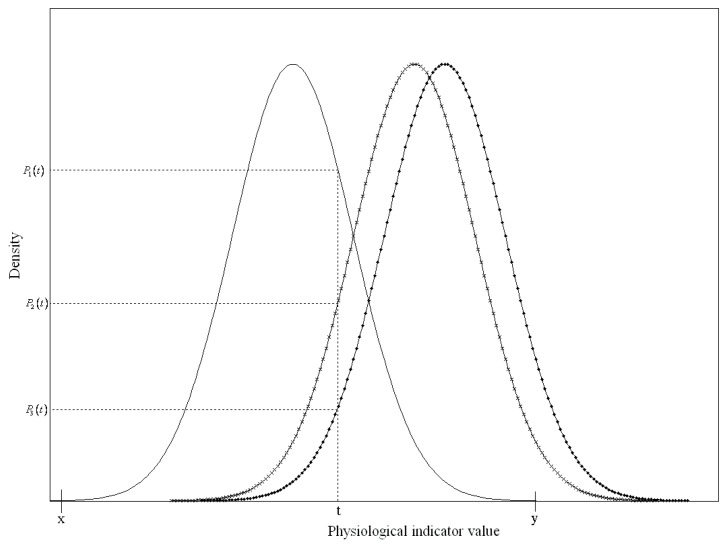
The relationship between probability density function and health risk index.

The R(t) was defined as a function of human health evaluation; it describes the *t* value of the risk factor once it has reached a certain value for a physiological state that was still healthy. In other words, R(t) is the health risk at value *t* for a particular risk factor. With an increase in the risk factor value, the disease or health risk will also increase. We assumed that *n* of multiple diseases were studied, and there was one normal state (healthy people) and ($2^{n}-1$) combinations of people who suffered from different diseases. The $f_{1,i}$ variable is representative of healthy individuals with the *i*^th^ risk factor in the probability density function. The $f_{2,i},f_{3,i},\ldots ,f_{2^{n},i}$ variables represent the probability density function of the different combinations of diseases with the *i*^th^ risk factor. The probability density function used the kernel density estimation approach. In this study, the health risk index $R_{i}\left(t\right)$ was defined for healthy people with the *i*^th^ risk factor and was calculated as follows:

(6) $$R_{i}\left(t\right)=\left\{ \begin{array}{ll} \frac{f_{1,i}}{f_{1,i}+f_{2,i}+\cdots +f_{2^{n},i}} & ,for\;x_{i}\leq t\leq y_{i}\\ 0 & ,others \end{array}\right.$$

where *t* represents the value of the *i*^th^ risk factor, and the interval $\left[x_{i},y_{i}\right]$ represents the range of values in the *i*^th^ risk factors for healthy people. Figure 3 presents an example of three different status functions of a risk factor to describe the health risk index. The solid line represents the function of the risk factor values of healthy subjects, and the other two lines with signs represent groups that suffered from different diseases. 

Since the *t* value of the risk factor only fell onto the *x* and *y*-axes for healthy subjects when it was between *x* and *y*, R(t) was the ratio of the probability density of the normal group $P_{1}\left(t\right)$ to all of the probability densities of the three status functions. Therefore, R(t) can be simplified as:

(7) $$R\left(t\right)=\frac{P_{1}\left(t\right)}{p_{1}\left(t\right)+p_{2}\left(t\right)+p_{3}\left(t\right)},\;\mathrm{for}\;x\leq t\leq y.$$

Each probability density value was a positive number less than 1. Therefore, R(t) was between 0 and 1.

If any of the risk factors were over the threshold values, we determined that the person had the disease(s). Therefore, the health risk index $R_{hri}\left(t\right)$ for a healthy person can be presented as follows:

(8) $$R_{hri}\left(t\right)=\overset{n}{\underset{i=1}{\Pi }}R_{i}\left(t\right)$$

**Figure 4:**
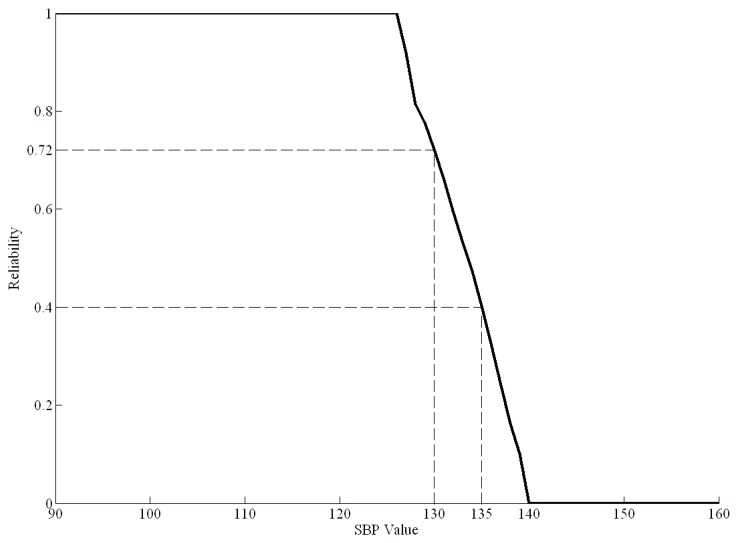
The health risk curve of systolic blood pressure (SBP).

We can plot the curve of the health risk index from $R_{hri}\left(t\right)$ for all *t* values of the risk factors. According to the health risk index curve, we can then evaluate the effect on health risks when risk factors change after a period of maintenance activities (such as exercise or diet control). Figure 4 shows an example of the health risk curve for SBP from 90 to 160 mmHg based on real data. 

In Figure 4, when the SBP is greater than 127 mmHg, the health risk curve begins to decline, indicating a gradual decline in health. When the SBP is greater than 140 mmHg, the health risk index is zero, indicating that the individual has the disease. For example, if a healthy person had an SBP of 135 mmHg before dietary control, the health risk index $R_{hri}\left(t\right)$ of SBP would be 0.4. Moreover, if their SBP decreased to 130 mmHg after a period of dietary control, the new health risk index $R_{hri}\left(t\right)$ of SBP would be 0.72. Therefore, the difference value between 0.4 and 0.72 (0.32) is the effect of the dietary control. 

## 3. Results and Discussion

Hypertension, hyperlipidemia, and hyperglycemia are three common diseases that are associated with metabolic syndrome and related to metabolic abnormalities. If a person has these diseases, the risk of developing other chronic diseases increases as well. A dataset from the National Health Insurance Research Database in Taiwan was used to elucidate the proposed analytical procedures. The dataset included 6496 subjects (3104 males and 3392 females) aged over 15 years old and 17 physiological indicators. 

Next, the analysis procedure established the appropriate kernel function for the common risk factors in every physiological state of hypertension, hyperlipidemia, and hyperglycemia. Table 2, Table 3, Table 4, Table 5 and Table 6 show the risk factors of the kernel density functions that were selected by the proposed estimation approaches used in this study. An example in Table 2 describes the results and indicates the outcome of the kernel density approach for FPG in the different physiological states (hypertension, hyperlipidemia, and hyperglycemia). In the normal state, the minimum value of the estimated six kernel density approaches was 0.0725. Therefore, the Gaussian type was selected as a suitable kernel function for FPG in the normal state. Similarly, the minimum value was 1.0622 for hyperlipidemia + hyperglycemia and 1.1331 for hypertension + hyperlipidemia + hyperglycemia. The Gaussian type was also the suitable kernel function for FPG in hyperlipidemia + hyperglycemia and hypertension + hyperlipidemia + hyperglycemia. The Triangular type was the appropriate kernel function for FPG for hypertension, hyperlipidemia, hyperglycemia, hypertension + hyperlipidemia, and hypertension + hyperglycemia, with the minimum values shown in Table 2. 

Based on the results in Table 2, Table 3, Table 4, Table 5, Table 6 and Table 7 summarizes all kernel density functions for the five common risk factors in every physiological state. 

**Figure 5:**
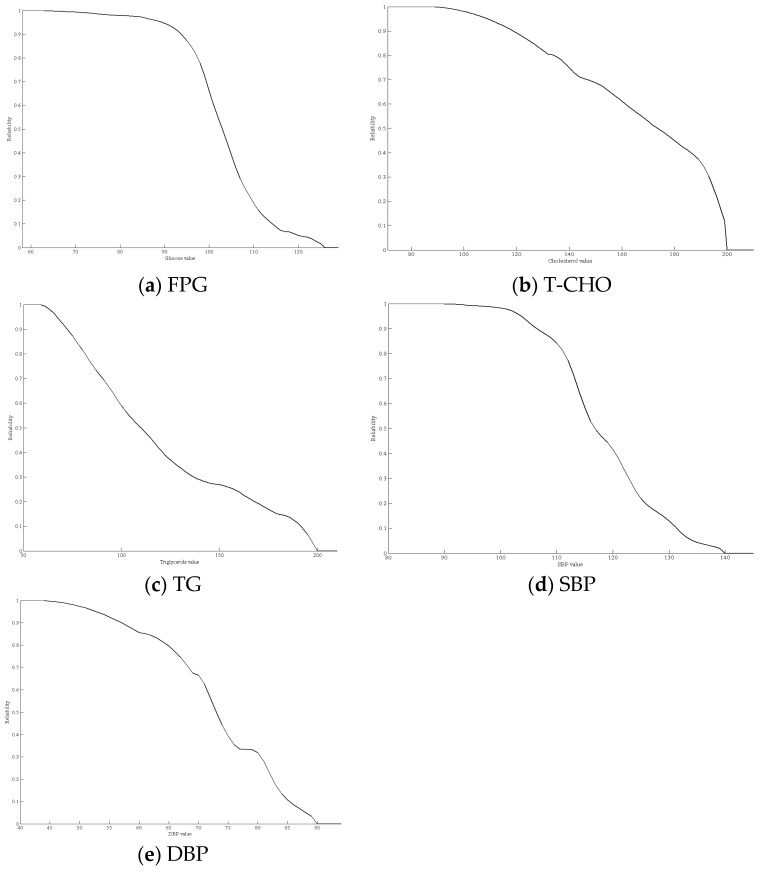
Health risk curves for the five risk factors.

Based on the data in Table 7 and the proposed formula, the health risk curve of the five risk factors in the normal state was calculated and plotted (Figure 5). Figure 5a–d displays the health risk curves for FPG, T-CHO, TG, SBP, and DBP. The health risk curves of the risk factors display monotonic decreasing curves, as shown in Figure 4. When the FPG reached 126 mg/dL, T-CHO or TG reached 200 mg/dL, SBP reached 140 mmHg, or DBP reached 90 mmHg, the health risk value became 0. When any risk factor value remained over the threshold, the probability that the subject was in the normal physical state was 0. The health risk index of the physiological system could be determined by multiplying the health risk values of the risk factors. Table 8 shows the results of ten subjects who were selected from the dataset.

Ten subjects were randomly recruited to measure the risk factor data. The brackets in Table 8 indicate the health risk index values. For example, the FPG value for subject 1 was 89 (0.9527); this indicated that the health risk index of an FPG level of 89 mg/dL was 0.9527. The health risk index of the physiological system of several subjects was 0 because some of the risk factor values were over the clinical thresholds, such as in Subjects 1, 2, 4, and 8. Subject 4 suffered from hypertension and hyperglycemia. The six healthy people were Subjects 3, 5, 9, 10, 7, and 6, arranged in descending order according to their health risk indexes. Although the six subjects had normal health statuses, the reliability values of the physiological systems differed. When applying the health index, the study of Stewart et al. [11] was used as a reference. Stewart et al. [11] simulated the effect of five risk factors after implementing an exercise regimen. After a six-week exercise routine, FPG, T-CHO, TG, SBP, and DBP levels decreased an average of 0.2 mg/dL, 5.2 mg/dL, 13.4 mg/dL, 5.3 mmHg, and 3.7 mmHg, respectively. We determined the change in the risk factors by calculating the health risk index. The results showed that subjects experienced a 7.29% reduction in their health risk index for hypertension, hyperlipidemia, and hyperglycemia.

The dataset on hypertension, hyperlipidemia, and hyperglycemia (Triple H) from the National Health Insurance Research Database in Taiwan and the research results of Stewart et al. [11] were used to explain the proposed methodology and application. Previous research has focused on disease prediction and medical treatment after disease identification. If judged as healthy, there is no corresponding early warning mechanism. The difference between the proposed method and the previous method is that this study develops an evaluation health risk index from the perspective of probability reliability. The estimation of the optimal probability distribution is established using the kernel density approach rather than using a normal probability function. The research results can be applied to solve the problem of health warning. When people are considered healthy, health risk indicators can be further calculated to understand their level of health. This benefits the individuals because risk factors can be controlled based on fitness by implementing strategies to increase exercise or control diet. Furthermore, the health risk index can be used to evaluate the effectiveness of the strategy and adjust the strategy. This will concretely improve the effective use of resources in the medical/health field. 

## 4. Conclusions

Based on the analysis of the results, we can make several conclusions regarding research in this area. The human body is a complex system, and a single-disease study is insufficient to assess multiple diseases. Most previous studies that conducted medical data analyses focused on risk factor selection and creating prediction and control models of diseases. These studies were able to monitor risk factors and estimate the risk of developing various diseases. However, the human body may not necessarily be completely healthy even when risk factors are all within normal ranges. Therefore, our study focused on assessing the degree of health. In this paper, common risk factors for multiple diseases were used to estimate the optimal probability density function, and a novel health risk index was proposed to evaluate the health status of healthy people. 

In practice, a health risk curve can be used to elucidate the relationship between risk factors and health risk. Prevention is often more desirable than curing diseases, and proper maintenance (such as participating in sports) may reduce the likelihood of further health decline. Furthermore, the effectiveness of preventive maintenance can be assessed through a health risk curve to determine appropriate adjustments to the maintenance strategy.

## Figures and Tables

**Table 1:** The common types of kernel function.

Types	Kernel Function
Uniform	$K\left(u\right)=\frac{1}{2}\;\mathrm{for}\;\left|u\right|\leq 1$
Triangular	K(u)=(1−|u|) for |u|≤1
Biweight	$K\left(u\right)=\frac{15}{16}{\left(1-u^{2}\right)}^{2}\;\mathrm{for}\;\left|u\right|\leq 1$
Epanechnikov	$K\left(u\right)=\frac{3}{4}\left(1-u^{2}\right)\;\mathrm{for}\;\left|u\right|\leq 1$
Gaussian	$K\left(u\right)=\frac{1}{\sqrt{2\pi }}e^{-\frac{1}{2}u^{2}}$
Cosine	$K\left(u\right)=\frac{\pi }{4}Cos\left(\frac{\pi }{2}u\right)\;\mathrm{for}\;\left|u\right|\leq 1$

**Table 2:** The selection of Kernel density function for the fasting plasma glucose (FPG).

FPG	Kernel Density Function					
	Gaussian	Epanechnikov	Uniform	Triangular	Biweight	Cosine
Normal	0.0725	0.0761	0.0781	0.0731	0.0753	0.0749
Hypertension	0.2387	0.2430	0.2508	0.2376	0.2425	0.2419
Hyperlipidemia	0.1242	0.1304	0.1374	0.1242	0.1286	0.1279
Hyperglycemia	0.9441	0.9485	0.9492	0.9428	0.9473	0.9468
(Hypertension & Hyperlipidemia)	0.2001	0.2052	0.2127	0.1995	0.2039	0.2033
(Hypertension & Hyperglycemia)	1.4843	1.4868	1.4935	1.4826	1.4863	1.4860
(Hyperlipidemia & Hyperglycemia)	1.0622	1.0658	1.0668	1.0624	1.0647	1.0643
(Hypertension & Hyperlipidemia & Hyperglycemia)	1.1331	1.1383	1.1439	1.1332	1.1367	1.1361

**Table 3:** The selection of Kernel density function for the total cholesterol (T-CHO).

T-CHO	Kernel Density Function					
	Gaussian	Epanechnikov	Uniform	Triangular	Biweight	Cosine
Normal	0.1184	0.1228	0.1281	0.1198	0.1214	0.1209
Hypertension	0.3555	0.3649	0.3781	0.3557	0.3613	0.3601
Hyperlipidemia	0.3247	0.3368	0.3404	0.3290	0.3328	0.3315
Hyperglycemia	0.8521	0.8631	0.8690	0.8532	0.8600	0.8587
(Hypertension & Hyperlipidemia)	0.4810	0.4864	0.4985	0.4796	0.4847	0.4841
(Hypertension & Hyperglycemia)	1.0488	1.0525	1.0584	1.0470	1.0515	1.0510
(Hyperlipidemia & Hyperglycemia)	0.9328	0.9400	0.9488	0.9331	0.9376	0.9368
(Hypertension & Hyperlipidemia & Hyperglycemia)	1.1214	1.1237	1.1235	1.1197	1.1230	1.1227

**Table 4:** The selection of Kernel density function for the triglyceride (TG).

TG	Kernel Density Function					
	Gaussian	Epanechnikov	Uniform	Triangular	Biweight	Cosine
Normal	0.1542	0.1569	0.1605	0.1542	0.1559	0.1555
Hypertension	0.4502	0.4506	0.4492	0.4490	0.4506	0.4506
Hyperlipidemia	0.3936	0.3973	0.4019	0.3945	0.3963	0.3959
Hyperglycemia	1.1723	1.1753	1.1776	1.1699	1.1745	1.1742
(Hypertension & Hyperlipidemia)	0.7481	0.7513	0.7497	0.7491	0.7504	0.7500
(Hypertension & Hyperglycemia)	1.4460	1.4466	1.4408	1.4404	1.4470	1.4470
(Hyperlipidemia & Hyperglycemia)	1.4483	1.4489	1.4411	1.4481	1.4487	1.4486
(Hypertension & Hyperlipidemia & Hyperglycemia)	1.5712	1.5751	1.5875	1.5712	1.5737	1.5732

**Table 5:** The selection of Kernel density function for the systolic blood pressure (SBP).

SBP	Kernel Density Function					
	Gaussian	Epanechnikov	Uniform	Triangular	Biweight	Cosine
Normal	0.1858	0.2023	0.2047	0.1914	0.1964	0.1947
Hypertension	0.4879	0.5139	0.5477	0.4952	0.5051	0.5021
Hyperlipidemia	0.2450	0.2562	0.2753	0.2474	0.2528	0.2519
Hyperglycemia	0.6924	0.7011	0.7258	0.6821	0.6983	0.6970
(Hypertension & Hyperlipidemia)	0.4440	0.4853	0.5307	0.4584	0.4709	0.4661
(Hypertension & Hyperglycemia)	1.1052	1.1400	1.1756	1.1091	1.1275	1.1232
(Hyperlipidemia & Hyperglycemia)	0.5720	0.5863	0.5930	0.5712	0.5811	0.5795
(Hypertension & Hyperlipidemia & Hyperglycemia)	0.8430	0.8601	0.8902	0.8378	0.8545	0.8523

**Table 6:** The selection of Kernel density function for diastolic blood pressure (DBP).

DBP	Kernel Density Function					
	Gaussian	Epanechnikov	Uniform	Triangular	Biweight	Cosine
Normal	0.2350	0.2585	0.2459	0.2463	0.2487	0.2459
Hypertension	0.5148	0.5454	0.5393	0.5201	0.5339	0.5295
Hyperlipidemia	0.2434	0.2656	0.2722	0.2501	0.2578	0.2557
Hyperglycemia	0.5583	0.5688	0.5843	0.5502	0.5657	0.5647
(Hypertension & Hyperlipidemia)	0.4065	0.4329	0.5135	0.4163	0.4276	0.4242
(Hypertension & Hyperglycemia)	0.9539	0.9985	1.0707	0.9558	0.9801	0.9745
(Hyperlipidemia & Hyperglycemia)	0.3873	0.4009	0.4333	0.3878	0.3962	0.3948
(Hypertension & Hyperlipidemia & Hyperglycemia)	0.6384	0.6800	0.7062	0.6471	0.6642	0.6591

**Table 7:** The suitable kernel function for five risk factors.

Kernel Function	Risk Factors				
	FPG	T-CHO	TG	SBP	DBP
Normal	Gaussian	Gaussian	Gaussian	Gaussian	Gaussian
Hypertension	Triangular	Gaussian	Triangular	Gaussian	Gaussian
Hyperlipidemia	Triangular	Gaussian	Gaussian	Gaussian	Gaussian
Hyperglycemia	Triangular	Gaussian	Triangular	Triangular	Triangular
(Hypertension & Hyperlipidemia)	Triangular	Triangular	Gaussian	Gaussian	Gaussian
(Hypertension & Hyperglycemia)	Triangular	Triangular	Triangular	Gaussian	Gaussian
(Hyperlipidemia & Hyperglycemia)	Gaussian	Gaussian	Rectangular	Triangular	Gaussian
(Hypertension & Hyperlipidemia & Hyperglycemia)	Gaussian	Triangular	Gaussian	Triangular	Gaussian

**Table 8:** The result of ten subjects for Health risk index.

Subjects	FPG	T-CHO	TG	SBP	DBP	Physiological State	Health Risk Index
1	89	177	153	141	90	Hypertension	0
	(0.9527)	(0.4751)	(0.2632)	(0.0000)	(0.0000)		
2	88	185	109	124	92	Hypertension	0
	(0.9579)	(0.4100)	(0.5070)	(0.2521)	(0.0000)		
3	86	119	56	97	60	Normal	0.7357
	(0.9672)	(0.8985)	(1.0000)	(0.9899)	(0.8552)		
4	238	190	103	140	69	(Hypertension & Hyperglycemia)	0
	(0.0000)	(0.3606)	(0.5591)	(0.0000)	(0.6752)		
5	80	143	53	133	67	Normal	0.0347
	(0.9793)	(0.7189)	(1.0000)	(0.0663)	(0.7432)		
6	105	167	99	92	60	Normal	0.1114
	(0.3917)	(0.5543)	(0.6012)	(0.9976)	(0.8552)		
7	79	169	117	101	73	Normal	0.1128
	(0.9800)	(0.5375)	(0.4378)	(0.9785)	(0.4997)		
8	97	234	263	112	80	Hyperlipidemia	0
	(0.8187)	(0.0000)	(0.0000)	(0.7724)	(0.3183)		
9	81	167	87	109	61	Normal	0.2926
	(0.9785)	(0.5543)	(0.7346)	(0.8627)	(0.8512)		
10	89	152	100	110	71	Normal	0.2005
	(0.9527)	(0.6766)	(0.5901)	(0.8425)	(0.6256)		
